# Fingerprint evidence in exoneration cases

**DOI:** 10.1016/j.fsisyn.2026.100675

**Published:** 2026-04-04

**Authors:** Simon A. Cole, Myleigh Schamp

**Affiliations:** Department of Criminology, Law & Society 2340, Social Ecology II University of California, Irvine, Irvine, CA, 92697-7080, USA

## 1 Introduction

In recent years, there have been a number of studies of the role of forensic evidence in wrongful convictions, e.g., [[1], [2], [3], [4], [5], [6], [7]], for a review, see [8]. Many of these studies use data from exonerations, cases in which a conviction was overturned because of new evidence of innocence. Such studies use exoneration data or some other source of cases to generate a data set of cases in which a forensic discipline falsely incriminated the defendant. We have used this approach ourselves in a study of cases in which fingerprint errors contributed to wrongful convictions that later resulted in exoneration [9].

Such studies exclude cases in which the forensic evidence was either: (1) not necessarily false; and/or (2) exculpated, rather than incriminated, innocent defendants. The approach of this study differs from all previous studies that use exoneration data to study forensic error, using fingerprint evidence as a convenient discipline on which to apply a broader approach.^1^ In this study, we examine all United States exoneration cases in which there was meaningful fingerprint evidence, regardless of whether it was erroneous and regardless of whether it incriminated the exoneree. This provides a broader look at the role of one forensic discipline in exoneration cases. Rather than asking how fingerprint error contributed to wrongful convictions, this study asks more broadly what role fingerprint evidence played in known exonerations. The answer is that it played a wide variety of roles, and in what follows, we try to make sense of them.

Consider the following two exoneration cases in which fingerprint evidence played a role:

*Lana Canen**and**Andrew Royer**were convicted in 2005 of the murder of a 94-year-old woman, Helen Sailor, who lived in their building in Elkhart, Indiana. A detective, Dennis Chapman, testified that Canen was the source of a fingerprint found on a plastic tub containing pill bottles in Sailor’s apartment. Other than the fingerprint, the evidence against Canen and Royer consisted of a statement by another resident of the building and a confession by Royer, a person with an intellectual disability. After conviction, an independent fingerprint expert, Kathleen Bright-Birnbaum, concluded that Canen was not the source of the print, and Chapman agreed he had made an error. Canen was exonerated in 2012, and Royer was exonerated in 2021.*

*Ignacio Dealba, Jr. was convicted of attempted murder in 2006 in Las Vegas after two men stole a bag of cash from a food truck worker and made their getaway in a red BMW. An off-duty police officer, Tim Shalhoob, gave chase, and one of the robbers shot at him from the BMW. The car was eventually traced to a man named James Vaughn, who named Dealba as his accomplice. Dealba was excluded as the source of 11 fingerprints found in the BMW. At trial, Shalhoob identified Dealba, who was Hispanic, as the robber despite having said earlier that the robber was a Black man. The conviction was reversed and then dismissed in 2009. In 2022, the Clark County District Court awarded Dealba a certificate of innocence based in part on an affidavit by Vaughn saying that the police pressured him to name an accomplice to “protect himself.”*

In both cases, the defendant(s) were innocent, and both are included in our data set. But the role of fingerprint evidence—and the conclusions that can be drawn from the cases—are quite different. The Canen case illustrates that fingerprint evidence can be mistaken or oversold. A wrongful conviction might have been averted if the jury had been more skeptical of fingerprint evidence in the face of the weakness of the other evidence. It cautions against the century-long efforts of the fingerprint examiners, prosecutors, and police to persuade the public to treat fingerprint evidence as “infallible.” The Dealba case is different. In that case, a wrongful conviction might have been averted had the jury given *more* weight to the fingerprint evidence, not less, especially in the face of the weakness of the other evidence. It cautions against giving undue weight to an eyewitness identification described by the court as “shaky at best” and the motivated testimony of a co-defendant when contradicted by forensic evidence.

This approach allowed us to answer the following research question:

• How many innocent defendants did fingerprint evidence tend to incriminate, how many did it tend to exculpate, and in what ways?

As we shall see, this approach also allowed us to answer two other research questions that arose adventitiously during the study:

• Are there “hidden” fingerprint errors that can be exposed by examining exoneration cases?
• To what extent did factfinders convict innocent defendants despite exculpatory fingerprint evidence?

## 2 Terms and definitions

In stating our research questions, we used a number of terms that require further explanation.

**Exonerations and innocence.** The data used in this study comes from the National Registry of Exonerations. Stated succinctly, the Registry defines exonerations as cases in which a person was convicted of a crime and later cleared of all charges based on new evidence of innocence. The Registry's detailed definition of exoneration is available online. The Registry's definition was devised with the aim of having a low number of inclusionary classification errors (i.e., factually guilty people included in the Registry) at the cost of a high number of exclusionary classification errors (i.e., factually innocent people excluded from the Registry) [10].^2^ When we describe exonerees as “innocent,” that is what we mean.

**Incriminate and exculpate.** We use the terms “incriminate” and “exculpate” to mean that generally the evidence tended to support the case for the defendant's guilt or innocence. In lay terms, we might say that incriminating evidence “helped” the defendant and exculpatory evidence “harmed” the defendant. In our first two stages of analysis, we use “incriminate” and “exculpate” in a fairly simplistic manner, e.g., identification of a defendant as the source of a questioned impression at a crime scene incriminates them, and exclusion of them as the source of that impression exculpates them. But source conclusions are neither incriminating nor exculpatory without context. An impression at a crime scene can be explained by legitimate access; an exclusion can be explained by a failure to leave an identifiable impression. In stage three of our analysis, we account for context in assessing the incriminating and exculpatory power of evidence.

**Consistent and Inconsistent.** Our data is not able to tell us “ground truth”—who was the source of the questioned impressions in the case. But Registry data can tell us whether expert opinions on that evidence remained consistent pre- and post-conviction, or whether they changed post-conviction. A “consistent” case was one in which expert authority consistently adhered to one conclusion (e.g., “identification” or “exclusion”) about the fingerprint evidence. This does not necessarily mean that conclusion was correct, but only that no expert authority ever opined that it was incorrect. An “inconsistent” case was one which at any point during the case an expert authority opined to any second conclusion. For example, the state's expert at trial opined that a comparison was “inconclusive,” but a review after trial opined that the comparison should have been called an “exclusion.” We do not arbitrate between these different opinions, but simply classify the fingerprint evidence in these cases as “inconsistent.“^3^

**Used.** We defined cases in which a fingerprint was “used” as cases in which there was not only a fingerprint present, but the fingerprint was examined and reported on in some way, whether pre- or post-conviction.

## 3 Data and materials

Cases used in this study are limited to those in which the exoneration occurred in 1989 or later. Convictions in these cases may have occurred in any year. The significance of the year 1989 is that it was the year of the first “DNA exoneration” in the U.S. The Registry, consistent with most wrongful conviction researchers, treats 1989 as the beginning of the “modern” era of exonerations, relevant to the working of the contemporary criminal legal system in a way that older cases may not be.

From this initial data set, we selected every case in which fingerprint evidence was used, a total of 218 cases, as of January 16, 2023, when the Registry contained a total of 3250 exoneration cases (see Supplementary Material 1). Although we use the common shorthand term “fingerprint,” we included palm or sole prints as well. The *exoneration case* served as the unit of analysis for this study, meaning cases in which a single fingerprint is “used” in the conviction of two or more people counts as two or more observations in our study if both of those people were convicted and subsequently exonerated. Similarly, a case in which fingerprint reports pertaining to multiple persons are used to convict a single person who is later exonerated counted as a single observation of the fingerprint report that was most incriminating to the exoneree.^4^

We serially consulted the following data from the Registry to understand the nature of the fingerprint evidence in the case: (1) the narrative summaries of each case^5^; (2) an internal Registry data field called “Describe Forensic Evidence”; (3) Registry source documents, primarily legal documents and media reports (trial transcripts are rare, laboratory reports even more so). We consulted as few sources as possible; only if the first source did not fully explain the nature of the fingerprint evidence, did we move on to the second source.

Our list of cases was generated by keyword searching the word “print” in both the Registry's narrative summaries and the Describe Forensic Evidence field. We then eliminated the large number of cases in which fingerprint evidence was not “used” according to the definition above. Therefore, our study is by no means a systematic analysis of the use of fingerprint evidence in 3250 cases. Instead, it is a study of cases in which the fingerprint evidence was important, or controversial, enough to merit mention in the sources we searched. We are, therefore, confident that there were “uses” of fingerprint evidence in the 3250 cases that we have missed and are not included in our study.

In most cases we lacked detailed information, such as trial transcripts, laboratory reports, or bench notes that would allow us a greater understanding of the treatment of fingerprint evidence in that case. In most cases, we coded the nature of the evidence based on a secondary source, such as a court's or litigant's characterization of the fingerprint examiner's conclusion. These characterizations, however, were often direct quotations or paraphrases. While litigants may have engaged in advocacy, they are unlikely to be wrong about basic facts such as what conclusion was selected or to whose known impression the questioned impression was compared.

## 4 Coding methods

### 4.1 Stage 1

**Fig. 1:**
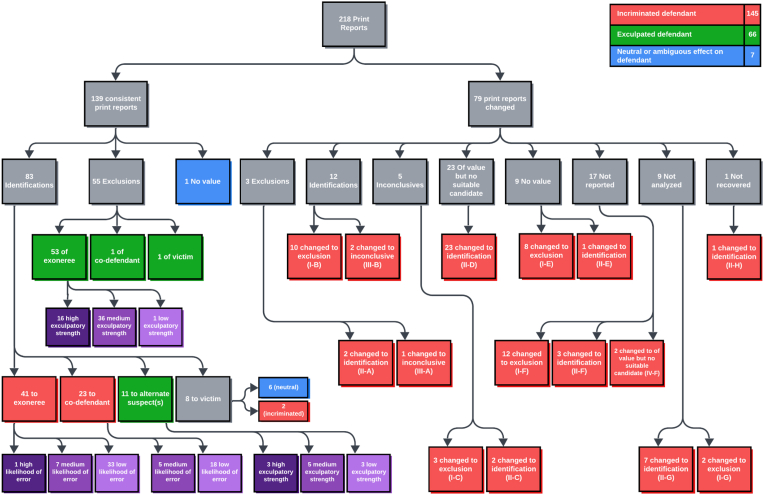
Complete flow chart of all 218 fingerprint reports.

Coding proceeded in three stages (see Fig. 1). In the first stage, we coded whether the fingerprint evidence in each of the 218 cases was “consistent” throughout the life of the case or “inconsistent.” Because these two categories raised different analytical questions, the “consistent” and “inconsistent” cases were subjected to different coding procedures in the second stage.

### 4.2 Stage 2

#### 4.2.1 Consistent cases

In the second stage, for “consistent” print reports, the conclusion of the friction ridge report was coded. Each case was assigned one of the four possible conventional conclusions used in the discipline during the period in which the cases occurred: (1) identification; (2) exclusion; (3) inconclusive; and (4) no value.^6^

The same fingerprint report (e.g., “identification”) can incriminate or exculpate the defendant depending on *to whom* the report pertains. For example, an identification report about a questioned impression from a crime scene that pertains to the defendant incriminates the defendant, but an identification report about that same impression that pertains to an alternate suspect exculpates the defendant. Therefore, we then coded which Person of Interest (POI) the report pertained to, if applicable (“no value” reports do not pertain to an individual). Each case was assigned to one of four POI codes: exoneree, co-defendant, alternate suspect, or victim.^7^

#### 4.2.2 Inconsistent cases

For inconsistent friction ridge reports, we coded the “error type,” which we defined as a combination of two variables: the initial fingerprint report and the final report. We used the matrix of error types proposed by [9], illustrated in Table 1.

**Table 1:** Matrix of fingerprint error types, discussed as “inconsistent” cases in this article. For example, a case in which an exoneree was convicted at a trial in which an examiner testified that a questioned impression had “no value,” but a post-conviction examiner said that, in fact, an alternate suspect had been identified as the source of that impression would be coded II-E, combining the post-conviction conclusion of “identification” with the pre-conviction conclusion of “no value.” “Consistent” cases would fall in the cells marked “Correct.” Source: [9].

			Final report				
			I	II	III	IV	V
			Exclusion	Identification	Inconclusive	Of value but no suitable candidate	No value
**Initial report**	**A**	**Exclusion**	CORRECT	Erroneous exclusion	Non-consensus exclusion	Erroneous exclusion	Erroneous exclusion
	**B**	**Identification**	Erroneous identification	CORRECT	Non-consensus identification	Erroneous identification	Erroneous identification
	**C**	**Inconclusive**	Non-consensus inconclusive	Non-consensus inconclusive	CORRECT	Non-consensus inconclusive	Non-consensus determination of value
	**D**	**Of value but no suitable candidate**	Failure to provide suitable candidate	Failure to provide suitable candidate	Non-consensus determination of value	CORRECT	Non-consensus determination of value
	**E**	**No value**	Non-consensus determination of no value	Non-consensus determination of no value	Non-consensus determination of no value	Non-consensus determination of no value	CORRECT
	**F**	**Not reported**	Failure to report	Failure to report	Failure to report	Failure to report	Failure to report
	**G**	**Not analyzed**	Failure to conduct an analysis	Failure to conduct an analysis	Failure to conduct an analysis	Failure to conduct an analysis	Failure to conduct an analysis
	**H**	**Not recovered**	Failure to recover probative evidence	Failure to recover probative evidence	Failure to recover probative evidence	Failure to recover probative evidence	Failure to recover probative evidence

### 4.3 Stage 3

In the third stage, we further coded two sets of “consistent” cases of particular interest. The first set was 64 cases with “consistent identifications” of the defendant or co-defendant. We coded these as to how likely it was that these identifications were erroneous, given that we now know the defendant was innocent. This was of interest because of the possibility that hitherto unknown erroneous identification occurred in some cases.

The second set was 53 cases with “consistent exclusions” of defendants. We coded the exculpatory strength of these fingerprint exclusions. This was of interest because some fingerprint exclusions may have been exculpatory, but we know that factfinders convicted defendants now known to be innocent in these cases.

## 5 Stage 1 results

Of the 218 friction ridge reports, 139 were consistent throughout the life of the case, and 79 were inconsistent (Fig. 1).

## 6 Stage 2 results

### 6.1 Consistent results

Of the 139 consistent reports, 83 were identifications, 55 were exclusions, and 1 was no value.^8^ Of the 83 consistent identifications, 41 identified the exoneree, 23 identified a co-defendant, 11 identified an alternate suspect or suspects, and eight identified the victim. Almost all (53/55) of the consistent exclusion reports excluded the exoneree from having been the source of the latent print. One excluded a co-defendant, and another one excluded the victim (see upper left of Fig. 1).

### 6.2 Inconsistent results

For “inconsistent” cases, the right side of Fig. 1 illustrates the conclusions of the initial print reports and the conclusions of the final print reports.

The most common error type present in inconsistent print reports, occurring in 23 cases (29% of the “inconsistent” cases) was II-D, or a missed identification resulting from the failure to provide a suitable candidate.^9^ One such case is that of James Blackmon, who was excluded as the source of a fingerprint found in the bathroom where the crime occurred. The source was not identified, and Blackmon was convicted of first-degree murder in 1988. Blackmon was exonerated in 2019 after examiners reported that a man named James Leach was the source of the print from the bathroom.

I-F, or a missed exclusion resulting from failing to report latent print evidence, was the second most common error type, occurring in 12 cases (15% of the “inconsistent” cases). The exoneration of Peter Dombrowski is an example of such cases:

*Dombrowski was convicted in 1985 of armed robbery, criminal use of a firearm, and vehicle theft and exonerated in 1990 after the Appellate Division of the New York Supreme Court ruled that Dombrowski had inadequate legal defense because his attorney failed to ask the prosecution to turn over evidence favorable to Dombrowski – namely, a fingerprint report that excluded him as a source of latent prints recovered from bottles touched by the perpetrator.*

Erroneous identifications, error types I-B and III-B, considered the most serious type of fingerprint error, occurred in a total of 12 cases.^10^

### 6.3 Effect of fingerprint evidence on exoneree

We then combined our analyses of “consistent” and “inconsistent” cases to determine in how many of the 218 cases the evidence incriminated or exculpated the exoneree.

The results are shown in Fig. 1. For “consistent” cases, “identification” reports that pertained to the exoneree or a co-defendant incriminated the exoneree. “Identification” reports that pertained to an alternate suspect exculpated the exoneree.

“Identification” reports that pertained to victims had to be further researched. In some cases, the identification of a victim's fingerprint in an incriminating location, such as the defendant's home or car, can be incriminating. But in other cases, the identification of a victim's fingerprint in a non-incriminating location, such as the victim's own home or car, has a “neutral” effect, neither incriminating nor exculpating the defendant. In 6 of the 8 “identification” cases that pertained to victims, the effect was neutral; in the two remaining cases the evidence incriminated the defendant.

“Exclusion” reports all exculpated the exoneree. Most exclusion reports pertained to the exoneree, but the one case in which it pertained to a co-defendant and the one case in which it pertained to a victim also exculpated the exoneree.

The one “no value” report case had a neutral effect.

For purposes of this study, we treat all “inconsistent” reports as incriminating the exoneree by denying them potentially exculpatory evidence and/or incriminating evidence implicating an alternate suspect.^11^

Summing the red cells in Fig. 1, we see that in two-thirds (145, or ∼67%) of the 218 cases the fingerprint evidence incriminated the exoneree. Summing the green cells, we see that in nearly a third (66, or ∼30%) of the 218 cases the fingerprint evidence exculpated the exoneree. Summing the blue cells, in only a small number (7, or ∼3%) of the 218 cases the fingerprint evidence had a neutral effect on the exoneree. Thus, overall, fingerprint evidence incriminated more innocent defendants than it exculpated. At the same time, it exculpated a significant number of innocent defendants. In those cases, fingerprint evidence supported the defendant's claims of innocence (which turned out to be correct), but juries convicted those defendants despite the fingerprint evidence.

#### 6.3.1 Incriminating evidence

Seventy-nine innocent defendants were incriminated by “inconsistent” evidence shown on the right branch of Fig. 1. In these cases, fingerprint evidence incriminated exonerees not only by implicating them through identification reports, but also by failing to exclude them or by failing to include alternate suspects. For detailed discussion and examples of how inconsistent print reports contributed to the conviction of innocent defendants, see [9].

Sixty-six innocent defendants were incriminated by “consistent” evidence shown on the left branch of Fig. 1. All but two of those 66 cases on the left branch were consistent identifications of the exoneree or a co-defendant. An example of how consistent identifications can incriminate defendants is found in the case of Austin Babb:

*On August 7, 2010, Demetrius Jones was murdered, and three others were wounded at a party in an apartment in the Bronx when a gunman entered and started shooting. Witnesses claimed to have seen the gunman enter the apartment building with another young man prior to the shooting and later saw them flee on bicycles. One witness stated that she saw one of the perpetrators crash their bicycle and subsequently flee on foot. Fingerprints found on the crashed bicycle were identified as coming from Babb and Wade Quiah, a member of the East Homicide Brims street gang. DNA was also found on the bicycle.*

*When police questioned Quiah, he denied involvement in the shooting and claimed to have left the party on a bicycle that crashed; after this, police stopped investigating Quiah and directed their attention solely to Babb. Babb was convicted due to the testimony of one eyewitness, Corey Collins, who admitted to drinking and smoking marijuana on the night of the shooting and pleading guilty to drug charges six months before the trial. Despite several other witnesses testifying that they did not see Babb at the party, Babb was convicted in 2014 and sentenced to forty-eight years in prison. In April 2016, the DNA profile from the bicycle was attributed to a man who had been arrested for defacing a firearm and had not been previously linked to the case. This DNA evidence, combined with serious inconsistencies in Collins’s testimony and the fact that the detective who conducted the eyewitness identification procedure had previously obtained false identifications in a separate case, undermined Babb’s conviction and led to his exoneration in 2020.*

The identification of Babb's fingerprint remains “consistent.” It was never rebutted or withdrawn. Was it erroneous? We don't know. All we know is that there was strong evidence of Babb's innocence—strong enough to persuade the court to vacate his convictions, and strong enough that revisiting the fingerprint identification was not legally necessary.

#### 6.3.2 Exculpatory evidence

As shown on the left side of Fig. 1, the two primary pathways through which fingerprint evidence exculpated exonerees was, first, consistent print reports which excluded the exoneree (53, or ∼80% of the 66 exculpatory cases); and, second, consistent reports that identified an alternate suspect (11, or ∼17% of the 66 exculpatory cases).

#### 6.3.3 Neutral effect on exoneree

Only 3% of print reports had a neutral or ambiguous effect on the defendant. Given our methods, discussed above, this is not surprising. Fingerprint evidence with a neutral effect on the probability of guilt or innocence is less likely to be mentioned, or mentioned prominently, in the source documents upon which the Registry relies. The Registry surely contains many cases in which fingerprint evidence had a neutral effect that are not included in our study. One case in which fingerprint evidence exerted a neutral effect can be seen in the case of Marvin Thomas:

*Marvin Thomas was convicted in 1987 of the murder of Janet Miller, based primarily on evidence that he had danced with Miller the night she died, on serology, and on microscopic hair comparison evidence. FBI fingerprint examiners reported that Miller was the source of questioned impressions found in her boyfriend Jeffrey Mosier’s car. Mosier was considered a suspect, but Thomas was prosecuted. Thomas’s conviction was overturned in 1992 because of careless handling of the forensic evidence, and he was acquitted at a second trial in 1993.*

The fingerprint evidence in this case – Miller's prints on Mosier's car (unsurprising, given their relationship) – provided neither exculpatory nor incriminating evidence against Thomas.

## 7 Stage 3 results

While [9] digs deeply into “inconsistent” report cases, this study presented us with an opportunity to delve deeper into “consistent” report cases. Because this study uses a unique data set of exoneration cases in which defendants can reasonably be presumed to have been innocent, it offered us opportunities to explore two further questions.

First, because exonerees are presumed innocent, consistent identifications of their fingerprints in an incriminating location raise the possibility that the identifications were erroneous, even in cases in which the identification has not been designated erroneous through consensus judgment. These would be hitherto unknown erroneous identifications. But in some cases, it may be possible that the exoneree was innocent, and the identification was still correct. To what extent were consistent identifications of exonerees and their co-defendants erroneous?

Second, because exonerees are presumed to be innocent, some cases raise the possibility that the factfinders disregarded exculpatory evidence. There are two types of cases in which this may have occurred: consistent identifications of alternate suspects and consistent exclusions of exonerees. These cases are intriguing because they may be cases in which factfinders were provided with strong exculpatory evidence and yet convicted the innocent defendant anyway. These may be cases in which greater deference to fingerprint evidence might have averted a wrongful conviction. But in some cases, because of the case facts, the evidence may not have been strongly exculpatory. In how many cases was exculpatory evidence given little weight by factfinders?

### 7.1 Potential erroneous identifications

We examined two sets of cases for potential erroneous identification: cases with consistent identifications to the exoneree and cases with consistent identifications to co-defendants.

#### 7.1.1 Consistent identifications of exonerees

Consistent identification reports that identify an exoneree as being the source of the latent print are intriguing. These are distinct from *erroneous identification* cases, in which, post-conviction, a report was rendered that the defendant was *not* the source of the questioned impression. In *consistent identification of exoneree* cases, no such post-conviction report was ever rendered. However, because, as noted above, we believe that exonerees are highly likely to be factually innocent, these cases suggest that an erroneous identification may have occurred. Because the report was “consistent,” if these were erroneous identifications, they were never named as such.

However, not every consistent identification report that identified the exoneree as the source of a print is necessarily an erroneous identification. Depending on the facts of the case, there may be possible or probable scenarios in which the exoneree is both factually innocent and the source of the latent print. To investigate this question, we assigned each case with a consistent identification report of the exoneree to one of three categories: high, medium, or low likelihood of error.^12^

After reading the cases, it became clear that the likelihood of error primarily depended on the question of access to the location from which the questioned impression was recovered (see Table 2). The results of this coding process are shown in the lower left corner of Fig. 1.

**Table 2:** Coding scheme for consistent identification reports that implicated the exoneree.

Likelihood of error	Definition
High	Defendant had no legitimate access to the location from which the latent print was recovered
Medium	Defendant might have had legitimate access to the location from which the latent print was recovered
Low	Defendant had legitimate access to the location from which the latent print was recovered

##### 7.1.1.1 High likelihood of error

We classified consistent identifications that identified the exoneree as having a high likelihood of error when the defendant had no legitimate access to the crime scene. We believe we have identified one such case (shown in dark purple in the lower left corner of Fig. 1) in which a hitherto unacknowledged erroneous identification likely occurred. This is the case of Jimmie Gardner who was falsely convicted of sexual assault in 1990 and exonerated in 2016:

*In May of 1987, a man broke into the home of B.F. and physically assaulted her and sexually assaulted her daughter, W.G. Around three months later, another woman, L.R. was sexually assaulted in her home. All three women lived in the Kanawha City section of Charleston, West Virginia, and all described their attacker as a Black man with a light or medium complexion, leading investigators to believe the same man committed both attacks. A fingerprint was found on a vase in B.F.‘s home. A rape kit was collected from W.G., and serological analysis indicated that the male portion was from a man with Type O blood. Black members of the Charleston Wheelers minor league baseball team were considered suspects, one of whom was Jimmie Gardner. Gardner’s fingerprints were taken, but, according to the police, they were never compared to the print from the vase.*

*Two years later, Gardner was arrested in Tampa, Florida and found in possession of a firearm. The firearm was traced back to Charleston, and the Tampa police notified the Charleston police. Detectives then submitted the print from the vase for fingerprint examination, and examiners reported that Gardner was the source of the fingerprint on the vase in B.F.‘s home. He was arrested and brought back to West Virginia. Gardner had no known connection to any of the three victims, nor did any of the victims identify Gardner as their attacker. Furthermore, Gardner had Type A blood. But forensic analyst Fred Zain, who would later be indicted for fraud, falsely told the jury that Gardner was not excluded as the source of the male portion of the rape kit, and Gardner was convicted of the rape of W.G., the assault on B.F., and robbery. Gardner was exonerated when Zain’s misconduct was exposed.*

Factually, we are left with three possible explanations for the consistent identification report:

1. The fingerprint report was correct, and Gardner was somehow guilty of the attacks on B.F. and W.G., despite not being the source of the male portion of biological evidence from the rape kit.
2. The fingerprint report was correct, and Gardner is innocent. Gardner's fingerprint ended up on the vase in some way other than by committing the crimes. Note that, as far as we know, even while being charged and tried and while litigating for post-conviction release, Gardner never offered any legitimate access explanation for his fingerprint being on the vase. At trial, Gardner was asked:
  Q: And you have no explanation for why your fingerprint was on this vase removed from the [B.F./W.G.] home, do you?
  A: I don't think my print was on that vase, sir, because I wasn't in that lady's house and, therefore, I know my print couldn't have been on that lady's vase because I wasn't there. …
  Q: If your fingerprint was lifted from this vase, you don't know how it got there, do you?
  A: That's correct, sir [11].
3. The fingerprint report was erroneous, whether intentionally or accidently.^13^

We find explanation (3) more plausible than either explanation (1) or (2) or their sum. We believe the identification of Gardner was likely erroneous, but a consensus judgment that the print was erroneous was never reached.

There are legal reasons why this consensus judgement is unlikely to ever be produced. Neither legal party has an incentive to investigate the matter. The State, having had one forensic analysis debunked already in this case, has little incentive to expose another. While Gardner might seem to have a greater interest in exposing the erroneous identification, it might not be in his interest to pursue the matter. Gardner has been exonerated. In the criminal matter, his position cannot be improved. So, the matter is likely to remain unresolved.

##### 7.1.1.2 Medium likelihood of error

We assigned seven cases to the second category, medium likelihood of error, in which the defendant might have had legitimate access to the crime scene. An example is the conviction of Tyrone Hood in 1996 for armed robbery and the first-degree murder of Marshall Morgan Jr., a 20-year-old basketball player at the Illinois Institute of Technology:

*Morgan borrowed his mother’s car for a date with his girlfriend on May 8, 1993. He did not come home, and on May 17, 1993, Morgan’s partially clad body was found wedged between the front and back seats of the car parked on a side street on the South Side of Chicago. The car contained a significant amount of trash.*

*Stanley Mocadlo and John Olejniczak, Chicago police officers assigned to the latent fingerprint unit, searched fingerprints recovered from two beer bottles found in the car in the Chicago Police Department Automated Fingerprint Identification System (AFIS) and concluded that 29-year-old Tyrone Hood, who lived about eight miles from where the body was found, was the source.*

*Attorneys for Hood discovered that Marshall Morgan Sr. had suddenly reappeared in his son’s life shortly before his murder and collected $44,000 on a life insurance policy taken out on Morgan Jr. Additionally, Morgan Sr.‘s late fiancé, Michelle Soto, had been found murdered in a similar manner as Morgan Jr., and Morgan Sr. had collected $107,000 from an insurance policy he took out on Soto. Despite this, Hood’s attorneys were not allowed to question Morgan Sr. about the life insurance policy on his son or on the policy and death of Soto.*

*Hood’s attorney, Jim Mullenix, argued that Morgan Sr. killed his son, put the body in the car, and threw bottles from a dumpster near Corliss High School, where Morgan Sr. worked as a janitor, on top of it. The high school was two blocks from Hood’s house.*

*However, in 2012, when the Cook County State’s Attorney Conviction Review Unit was considering his case, “Hood said he had no explanation for his fingerprints on the beer bottles”* [12].

*In July 2014, the* New Yorker *magazine published an article outlining all the evidence that Morgan Sr. killed his son* [13]*, and in November 2014, lawyers for Hood filed a clemency petition. They argued that the evidence against Hood was based on testimony that had been recanted, and they pointed to Morgan Sr.‘s sudden reappearance in his son’s life after being absent for most of his life and the insurance policy he took out on his son’s life. Hood was exonerated in January of 2015.*

We code this case as “maybe erroneous” because Mullenix offered a plausible explanation for the fingerprint identification being accurate that is also consistent with Hood's innocence. Mullenix's explanation seems as plausible as the alternate hypotheses: first, that the fingerprint identification was erroneous and, second, that Hood was guilty.

##### 7.1.1.3 Low likelihood of error

We coded the remaining 33 cases as having a low likelihood of error. The most common scenario for these cases was that the defendant indisputably had legitimate access to the crime scene. One case that falls in this category is that of Alan Beaman, a man charged with the murder of his former girlfriend, Jennifer Lockmiller, in 1995 and exonerated in 2009:

*In August of 1993, Lockmiller was found dead in her apartment in Normal, Illinois, after being strangled with a clock radio cord and stabbed in the chest with a pair of scissors. Fingerprints recovered from the clock radio were attributed to Lockmiller’s current boyfriend, Michael Swaine, her ex-boyfriend, Beaman, and an unidentifiable source. Beaman said he often used the clock radio when he was dating Lockmiller. The relationship had ended in Spring 1993.*

Our assumption of Beaman's innocence is consistent with his being the source of the latent prints on the clock radio. We assume fingerprint errors are at least somewhat rare and that the leaving of fingerprints in an apartment where one is sleeping is common. It seems more likely that Beaman was innocent of the murder and left the mark on the clock radio at another time than that the attribution of the mark to Beaman was erroneous.

##### 7.1.1.4 Summary

Of the 41 consistent identifications that identified the exoneree, only one identification was categorized as having a high likelihood of error. This case, the Jimmie Gardner conviction, has never been described as an erroneous identification case. Nonetheless, we would argue for considering this case when considering the population of known erroneous identifications. In addition, we identified seven other cases in which we think an erroneous identification may have occurred. Researchers may want to consider these cases when considering instances of erroneous identification.

#### 7.1.2 Consistent identifications of Co-defendants

Consistent identification reports that identify an exoneree's co-defendant as being the source of the latent print represent a peculiar situation. Unlike consistent identification reports that identify the exoneree, as discussed in the previous section, consistent identification reports that identify the exoneree's co-defendant serve to implicate the exoneree when the prosecution uses the print report to connect the co-defendant's supposed guilt to the exoneree's. There are thirteen such cases present in our dataset.

To shed light on other potential erroneous identifications, we applied a similar coding procedure as that used in section 7.1. Unlike consistent print reports which identified the exoneree, the likelihood of error for consistent identifications to co-defendants depends on both the co-defendant's access to the crime scene and how “tightly coupled” [14] the exoneree and co-defendant were (i.e., how dependent the innocence or guilt of one is on the innocence or guilt of the other) (Table 3). The results are shown on the lower left of Fig. 1.

**Table 3:** Coding scheme for consistent identification reports that implicated the co-defendant.

Likelihood of error	Definition
High	Co-defendant and defendant were tightly coupled, and co-defendant had no legitimate access to the crime scene
Medium	Co-defendant might have had legitimate access to the crime scene
Low	Co-defendant had legitimate access to the crime scene

##### 7.1.2.1 High likelihood of error

We found no cases that fell in this category.

##### 7.1.2.2 Medium likelihood of error

In five cases, the co-defendant might have had access to the crime scene. One example is the case of Sean Ellis:

*Ellis was convicted in 1995 of the 1993 murder of John Mulligan, an off-duty Boston police officer who was shot in his car while providing security at a Walgreens drug store. Seventeen prints were lifted from Mulligan’s vehicle. One man who lived on a residential street near the Walgreens claimed to have seen two men get out of a brown Volkswagen Rabbit at 3:20 a.m., and that a woman remained in the back seat.*

*Around a week after Mulligan’s murder, detectives found a brown Volkswagen matching the description given by the witnesses and tracked the vehicle identification number to Mark Evans, who claimed that eighteen-year-old Terry Patterson was the primary driver of the car.*

*Patterson and Ellis said Patterson drove Ellis to the Walgreens to buy diapers for Ellis’s cousin. They stopped by a payphone to call a friend, purchased the diapers, and went home. Both Patterson and Ellis were arrested on charges of first-degree murder.*

*The primary evidence against Patterson was the fingerprint testimony, during which the fingerprint examiner stated that the impressions taken from the exterior driver’s side door of Mulligan’s vehicle consisted of four “simultaneous” impressions – made by four fingers from the same hand at the same time (a method that was not the standard method for fingerprint identification) – were left by Patterson. The primary evidence against Ellis was the dubious identification by an eyewitness, who had connections to one of the detectives who conducted the lineups. Patterson and Ellis were both convicted and sentenced to life in prison.*

*In 2000, the Supreme Judicial Court of Massachusetts reversed Patterson’s convictions, and in 2005, in a later appeal, the court ruled that the “simultaneous” fingerprint evidence against Patterson would not be allowed during his retrial. Patterson pled guilty to manslaughter in 2006 and was released in 2007 after receiving credit for time served. A new trial was granted for Ellis in 2015 because the prosecution and police had failed to disclose evidence of other possible suspects and the detectives involved in Mulligan’s case had been indicted on numerous corruption charges. In 2018, Ellis’s convictions were vacated.*

This case is categorized as medium likelihood of error because it is possible that Patterson did indeed touch Mulligan's car at some point while Ellis was purchasing diapers in the Walgreens.

##### 7.1.2.3 Low likelihood of error

Eighteen cases were categorized as low likelihood of error on the grounds that the co-defendant indeed had legitimate access to the crime scene. One of these cases is that of Quedillis Ricardo Walker and unexonerated co-defendant Rahsson Bowers:

*In 1991, thirty-four-year-old Lisa Hopewell was discovered bound, suffocated with duct tape, and stabbed in the guest bedroom of her apartment in Cupertino, California. Thirty-one questioned impressions were collected from the crime scene; fingerprint examiners reported that Bowers, a drug dealer, was the source of the questioned impressions. Bowers denied any involvement in Hopewell’s murder until police informed him that his fingerprints were found on the duct tape, after which Bowers implicated himself and Walker, who had a previous romantic relationship with Hopewell. Walker denied any involvement in the crime and stated that he was at a motel with Jacqui Miller on the night of the murder. Miller, however, denied that she was with Walker when questioned by police. Walker and Bowers were subsequently arrested and charged with murder.*

*Bowers pled guilty to second-degree murder and agreed to testify against Walker. Walker was convicted of first-degree murder and was sentenced to twenty-six years to life in prison.*

*After his conviction, Walker hired a new defense attorney, who found five witnesses who testified that Walker was not at the murder scene. Additionally, several witnesses were found who identified another man, Mark Anthony Swanson, as Bowers’s accomplice in Hopewell’s murder, and DNA testing on cigarette butts found at the scene was consistent with him. In 2003, Walker was exonerated after the Santa Clara County District Attorney’s office conducted its own re-investigation.*

We categorized this case as low likelihood of error because Bowers had legitimate access to Hopewell's apartment due to their drug-related interactions.

### 7.2 Potential exculpatory evidence

There were two scenarios in which fingerprint evidence may have presented exculpatory evidence in cases in which factfinders nonetheless decided to convict.

#### 7.2.1 Consistent identification of alternate suspects

In 11 cases, there was a consistent identification of an alternate suspect. However, we cannot assume that the exculpatory strength of these identifications was high in all of these cases. We coded the 11 cases according to whether the exculpatory strength of the identification to the alternate suspect was high, medium, or low (Table 4). The results are shown in the bottom center of Fig. 1.

**Table 4:** Coding scheme for consistent identifications of alternate suspects.

Exculpatory Strength of Identification of Alternate Suspect	Definition
High	Print in location likely to be accessible to true perpetrator and few or no others
Medium	Print in location proximate to, but not directly related to, crime
Low	Print location consistent with prosecution's theory of exoneree's guilt

##### 7.2.1.1 High exculpatory strength

In three cases, the identification to the alternate suspect had high exculpatory strength. An example is the conviction of Dewey Bozella for the murder of an elderly woman in her apartment in New York in 1983. Fingerprint examiners reported that the alternate suspect, Donald Wise, was the source of a print found on the inside of the bathroom window of the apartment. “Although the fact that Wise's fingerprint was located inside Emma Crapser's apartment was brought out at trial, along with Wise's” conviction for a similar murder of an elderly woman, “the jury, nevertheless, rejected Wise as the killer” and convicted Bozella [15].

##### 7.2.1.2 Medium exculpatory strength

In five cases, the identification to the alternate suspect had medium exculpatory strength. An example is the conviction of Richard Burkhart for murder in Montana in 2002. Fingerprint examiners reported that two alternate suspects were the sources of prints found on Burkhart's car near the murder scene. The prints incriminated the two alternate suspects in having robbed Burkhart on the night of the murder, but they did not directly implicate them in the murder.

##### 7.2.1.3 Low exculpatory strength

In three cases, the identification to the alternate suspect had low exculpatory strength. An example is the conviction of Frances Choy for murder in Massachusetts in 2011. Fingerprint examiners reported that the alternate suspect (Choy's nephew) was the source of fingerprints found on notes outlining the plan for the crime. But the prosecution's theory of the crime was that Frances acted in concert with her nephew, so the identification of her nephew's prints had little exculpatory strength.

#### 7.2.2 Consistent exclusions of exonerees

Another intriguing set of cases are the 53 cases with consistent exclusion reports that excluded the exoneree as being the source of the latent print (cell I-A of Table 1). We cannot assume that the exclusions were highly exculpatory in all 53 cases because in some of them the evidence may not have been very exculpatory or even exculpatory at all. The exculpatory strength of a fingerprint exclusion varies depending on the facts of the case. The exclusion of a defendant as the source of a print in blood on the wall of a home to which the defendant has no legitimate access is stronger exculpatory evidence than their exclusion as the source of print found on the exterior of a car in a public place. Again, we need to take a closer look at the fact pattern of each case to determine how probative of innocence the consistent exclusion evidence was.

Upon reading the cases, it became apparent that the crucial issue in assigning these probabilities was the strength of the association between the questioned impression and the crime. Some questioned impressions are highly associated with the crime (e.g., a print in blood on the murder weapon); others much less so (e.g., a print on the outside of a car). To make this assessment, we again assigned each case an exculpatory strength of high, medium, or low (Table 5). The results are shown in the middle left of Fig. 1.

**Table 5:** Coding scheme for consistent exclusions that excluded the exoneree.

Exculpatory strength of exclusion	Definition
High	Highly likely that the print is from the true perpetrator (e.g., on murder weapon, in blood, on object perpetrator touched)
Medium	Print is from somewhere in the crime scene
Low	Print is not necessarily from true perpetrator

##### 7.2.2.1 High exculpatory strength

We found 16 such cases. The case of Timothy Bridges is an example:

*Bridges was convicted in 1991 in North Carolina for sexual assault of a woman in her home. The police said three informants had reported that Bridges had admitted to the crime. While processing the crime scene, a technician found a bloody palm print on a wall in the back of the bedroom adjacent to a light switch. Bridges’s attorney argued that a bloody palm print found on a wall in the victim's home was left by the perpetrator. Bridges and the victim were excluded as the source of the print. On February 2, 1991, the jury convicted Bridges of all charges. He was sentenced to life in prison.*

Since it is highly likely that the print found on the wall was left by the true perpetrator, the exculpatory strength of this exclusion report was high for Bridges, but the jury convicted him nonetheless.

##### 7.2.2.2 Medium exculpatory strength

We found 36 such cases. For example, consider the case of Mallory Nicholson:

*Nicholson was convicted in 1982 in Dallas, Texas for sexual assault of two boys. Nicholson was accused of luring the boys to help him break into an apartment where he stole items and assaulted the boys. A fingerprint was found on the toilet tank, but Nicholson was excluded as the source. Nicholson was convicted based primarily on the victims' identification of him and sentenced to 55 years in prison. In 2021, the Texas Court of Criminal Appeals granted Nicholson’s habeas corpus petition based on evidence that the prosecution had concealed exculpatory evidence that implicated another suspect. Whether the fingerprint was ever compared to that suspect is not known. The District Attorney dismissed the case in 2022.*

Since the questioned impression on the toilet tank was in the burgled apartment it may have been from the true perpetrator, but it also may have been unrelated to the crime. Thus, the exculpatory strength of the exclusion report was categorized as medium.

##### 7.2.2.3 Low exculpatory strength

The only case that fell in this category was that of Timothy B. Cole, a man wrongly convicted of the rape of Michelle Mallin in 1986:

*Police believed the man who assaulted Mallin might have been an unknown serial rapist known at the time as the “Tech Rapist” who was thought to be tied to four other attacks. Despite this suspected connection, Cole was never charged with committing any other rapes besides Mallin’s. Cole and his attorney attempted to present evidence that a very similar attack had occurred one month before the assault of Mallin and that Cole had been excluded from being the source of fingerprints from the victim’s car; however, the judge would not allow this evidence to be presented to the jury.*

The exculpatory strength of this exclusion is low because it is possible that the prints left in the victim's car were not from the same perpetrator who committed Mallin's rape (despite similarities between the two crimes), or the prints could have been unrelated to the crime. Therefore, excluding Cole as being the source of those prints only provided weak evidence against the charges in the attack on Mallin.

##### 7.2.2.4 Summary

Factfinders convicted defendants now known to be innocent in 60 cases in which there was exculpatory evidence with high or medium strength. These included 52 cases in which the defendant was excluded as the source of a questioned impression and 8 cases in which an alternate suspect was identified as the source of a questioned impression. In 19 of these 60 cases, the exculpatory strength was high: 16 exclusions of the exoneree and 3 identifications of alternate suspects. This exculpatory evidence did not persuade factfinders not to convict even defendants we now know to be innocent. This finding seems to support mock jury studies which suggest that factfinders tend to undervalue fingerprint exclusions [16].

To be sure, the exculpatory strength of fingerprint exclusions has inherent limitations. Absence of evidence is not evidence of absence: the exclusion of a person as the source of an impression only means that person is not the source of *that* impression. It does not necessarily mean that person didn't touch that surface. And, it does not necessarily mean that the source of *that* impression was the perpetrator of the crime, as opposed to someone unrelated to the crime. Because it is not possible to time-date fingerprints, in most cases unidentified crime scene impressions could, in principle, have been deposited at any time and therefore not be from the true perpetrator of the crime. The assumption that an unidentified crime scene impression derives from the true perpetrator may also depend on the degree to which “elimination” prints (i.e., known prints from persons known to have legitimate access to the scene) have been compared.

## 8 Conclusion

Fingerprint evidence is powerful. It enjoyed nearly a century of calling itself “infallible”—and having that claim taken seriously by courts and by the public. More than 20 years ago, when the fingerprint community was still claiming infallibility, this point was most poignantly illustrated by Stephan Cowans, who, after being convicted based on an erroneous fingerprint identification, said “that if he had been on the jury, he would have voted to convict himself” [17]. The exposure of erroneous identifications, some in exoneration cases, some not, eventually eroded that claim, showing that fingerprint evidence was capable of incriminating and convicting the innocent. In a separate study, we showed that fingerprint evidence incriminated innocent defendants in a variety of other ways too, such as by calling “inconclusive” comparisons that should have excluded defendants [9].

We highlight cases in which the fingerprint evidence was not sufficient to lead jurors to the correct decision, even when fingerprint evidence provided exculpatory information for innocent defendants. While incriminating fingerprint evidence has contributed to many false convictions, suggesting that juries should check their trust in fingerprint evidence, in many other cases, false convictions might have been avoided had juries given greater weight to fingerprint evidence.

This damned-if-you-do, damned-if-you-don't picture may be discouraging for forensic examiners and criminal defendants alike. While even erroneous or sloppy fingerprint evidence helped convict, even exculpatory fingerprint evidence failed to acquit. Presumably, the exculpatory forensic evidence could not overcome the other evidence or the conviction-proneness of most juries.

In short, while some innocent defendants would have done better with less weight accorded to fingerprint evidence, others would have done better with more. Innocent defendants, it seems, need not more fingerprint evidence, nor less, but better.

## CRediT authorship contribution statement

**Simon A. Cole:** Writing – review & editing, Writing – original draft, Methodology, Investigation, Formal analysis, Data curation, Conceptualization. **Myleigh Schamp:** Writing – review & editing, Writing – original draft, Visualization, Software, Formal analysis.

## Declaration of competing interest

The authors declare that they have no known competing financial interests or personal relationships that could have appeared to influence the work reported in this paper.

## Footnotes

13 It is known that Zain handled the vase. And, the police claim that they did not compare the print from the vase to Gardner's known prints until his arrest in Tampa, two years after they obtained his prints.

## Footnotes

1 Although “friction ridge” is currently the preferred technical term among experts, for the benefit of its broad readership, this article will use the common term “fingerprint.”

2 In this study, we assume the factual innocence of all defendants in the data set. We consider this assumption true in a statistical sense – that is, we assume that the Registry contains a small number of random or systemic errors and that in a small number of cases our assumption of innocence will be false. Consistent with the nature of scientific measurement error, *which* specific observations (or cases) have been erroneously classified is, of course, not known to us. Despite these assumptions, we refer to all exonerees as “innocent” for clarity of discussion.

3 “Inconsistent” cases include those in which the original examiner made a non-consensus determination (e.g., identifying the exoneree as being the source of the latent print when other examiners concluded the exoneree should instead be excluded). Additionally, inconsistent reports include cases in which it appeared to the defense that there was no print report whatsoever when in fact the print report or print evidence itself was suppressed by the prosecutor, thus denying the defense from using the print report during the exoneree's original trial. Inconsistent reports also include cases in which the examiner failed to conduct an examination altogether.

4 We made one exception to this rule in the case of Douglas Warney in order to make the data in this report consistent with the data in [9]. Warney is counted twice: for the comparison to Warney, the exoneree, and for the comparison to the alternate suspect.

5 Narrative summaries are written by Registry personnel and are published for each case on the online archive. The qualifications of the authors have changed over the history of the Registry and include students, innocence organizations, professional journalists, and academic editors. The majority of Registry summaries have been written by professional journalists. In this article, the capsule case summaries were written by the article authors based on the narrative summaries.

6 The fingerprint discipline's adherence to this “categorical” approach to reporting results, using only four possible conclusions, is currently in the process of changing. However, this is a recent development, and it is not known to have affected any of the cases in this study.

7 There were print reports in which the evidence pertained to multiple individuals. In such cases, we coded the report that was most probative of innocence for the exoneree. For instance, if a report identified the impressions of both the victim and an alternate suspect at a crime scene, we coded the case as an identification to an alternate suspect since identifying the victim's impressions at the crime scene does little to help exculpate the defendant.

8 Readers should not form the impression that this distribution of conclusions is at all representative of the distribution of conclusions that might be seen in an analysis of all examination results in a fingerprint examination unit. The information that the Registry uses is likely to be skewed toward conclusions that are consequential to the case, such as “identification” and “exclusion,” rather than “inconclusive” and “no value.” For a study with a representative distribution from a fingerprint examination unit, see [18].

9 The enigmatic term “failure to provide a suitable candidate” is one that we borrowed from the Scientific Working Group for Friction Ridge Analysis Study and Technology (SWGFAST) [19]. Most commonly it refers to a scenario in which pre-conviction fingerprint analysts were not able to find a suitable candidate source for a questioned impression. However, post-conviction, they were able to find a suitable candidate source, usually through the use of a database search. In other words, the “change” in these cases was driven by the use of a database. This type of “error”—and whether indeed they should even be called “errors”—is discussed further in [9].

10 The 12 cases include 10 cases (including Lana Canen, Stephan Cowans, Beniah Dandridge, Dennis Fritz, Richard Jackson, Andrew Royer, and Ronald Williamson) in which the post-conviction consensus view was “exclusion” (error type I-B in Table 1). Three of the 10 (Shirley Kinge, William LaBolt, Jr.,Mark Prentice) are cases that involved deliberate fabrication of fingerprint evidence by New York State Trooper David Harding. We would not include these three cases in a study of “fingerprint errors” that is aimed at measuring the prevalence of unintentional fingerprint errors, e.g., [20]. But this is not a study of fingerprint error, intent is not at issue, and for the purposes of this study they are correctly described as erroneous identifications. Other cases, such as the Cowans case, may or may not have been intentionally erroneous identifications. The 12 cases also include 2 cases (Clemente Aguire-Jarquin and Kevin Siehl) in which the post-conviction consensus view was “inconclusive” (error type III-B in Table 1).

11 For discussion of why we perceive all inconsistent reports as incriminating defendants, see [9].

12 All “high, medium, low” scales used in this paper are intended as ordinal scales. Thus, the term “high” has no inherent meaning other than relative to the other categories on the scale.

## References

[1] Garrett B.L., Neufeld P. (2009). Invalid forensic science testimony and wrongful convictions. Va. Law Rev..

[2] Cole S.A., Meterko V., Chu S., Cooper G., Paredes J.W., Possley M., Otterbourg K. (2022).

[3] Cole S.A., Paredes J.W., Possley M., Otterbourg K. (2023). Microscopic hair comparison analysis and convicting the innocent. Nat. Regist Exonerat..

[4] Cole S.A., Possley M., Otterbourg K., Paredes J.W., O'Brien B., Cousino M., Gross S.R. (2024).

[5] Morgan J. (2023). Wrongful convictions and claims of false or misleading forensic evidence. J. Forensic Sci..

[6] Smit N.M., Morgan R.M., Lagnado D.A. (2018). A systematic analysis of misleading evidence in unsafe rulings in England and Wales. Sci. Justice.

[7] LaPorte G.M. (2017).

[8] Bonventre C.L. (2021). Wrongful convictions and forensic science. WIREs Forensic Sci..

[9] Cole S.A., Scheck B.C. (2017/2018). Fingerprints and miscarriages of justice: ‘Other’ types of error and a post-conviction right to database searching. Alb. L. Rev..

[10] Gross S.R., Shaffer M. (2012).

[11] State v. Gardner (1990). Criminal No. 89-F-153 Tr. trans..

[12] Mills S. (2012).

[13] Schmidle N. (2014).

[14] Perrow C. (1984).

[15] Bozella People v. (2009).

[16] Garrett B.L., Mitchell G. (2013). How jurors evaluate fingerprint evidence: the relative importance of match language, method information, and error acknowledgement. J. Empir. Leg. Stud..

[17] Thomas J. (2004).

[18] Rairden A., Garrett B.L., Kelley S., Murrie D., Castillo A. (2018). Resolving latent conflict: what happens when latent print examiners enter the cage?. For. Sci. Int..

[19] SWGFAST (2012). Scientific Working Group on Friction Ridge Analysis Study and Technology.

[20] Cole S.A. (2005). More than zero: accounting for error in latent fingerprint identification. J. Crim. Law Criminol..

