# Corrigendum to “Fingerprint evidence in exoneration cases” [Forensic Science International: Synergy 12 (2026) 100675]

**DOI:** 10.1016/j.fsisyn.2026.100683

**Published:** 2026-05-09

**Authors:** Simon A. Cole, Myleigh Schamp

**Affiliations:** Department of Criminology, Law & Society 2340, Social Ecology II University of California, Irvine, Irvine, CA, 92697-7080, USA

The authors regret that the Funded details was not included in the published article. The funded statement is provided below:

“This research was funded in part by the Center for Statistical Applications in Forensic Evidence sponsored by the National Institute of Standards and Technology [70NANB15H176]."

The authors would like to apologies for any inconvenience caused.

