# Supplementary material for: Fingerprint evidence in exoneration cases
Source: Forensic Sci Int Synerg. 2026 Apr 4;12:100675. doi: 10.1016/j.fsisyn.2026.100675 (PMC13090315; doi:10.1016/j.fsisyn.2026.100675)
Supplement: Multimedia component 1 [file mmc1.pdf]

Supplementary Material 1: Table of 218 cases analyzed in this study.

Last Name	First Name	Exoneration Year	Const. Report?	Error Type (if inconst.)	POI *Final* Report Pertained to (if inconst.)	Report Type (if const.)	POI Report Pertained to (if const.)	Likelihood of erroneous identification (if appl.)	Exculpatory strength (if appl.)	Effect?
Abrego	Eruby	2022	Y	n/a	n/a	Exclusion	Exoneree	n/a	Medium	
Aguirre-Jarquin	Clemente	2018	N	III-B	Exoneree	n/a	n/a	n/a	n/a	
Allen	Dennis	2019	Y	n/a	n/a	Exclusion	Exoneree	n/a	High	
Allen	Billy Frederick	2011	Y	n/a	n/a	Identification	Exoneree	Unlikely	n/a	
Allen	Christopher	2006	Y	n/a	n/a	Identification	Exoneree	Unlikely	n/a	
Allen, Jr.	George	2013	N	I-E	Exoneree	n/a	n/a	n/a	n/a	
Andrews	Isaiah	2021	N	I-F	Exoneree	n/a	n/a	n/a	n/a	
Armstrong	LaMonte	2013	N	II-A	Alternate Suspect(s)	n/a	n/a	n/a	n/a	
Aziz	Muhammad	2021	Y	n/a	n/a	Identification	Co-defendant	Unlikely	n/a	
Babb	Austin	2020	Y	n/a	n/a	Identification	Exoneree	Maybe	n/a	
Bailey	Kevin	2018	Y	n/a	n/a	Exclusion	Exoneree	n/a	High	
Baker	Dontrell	1997	Y	n/a	n/a	Identification	Co-defendant	Unlikely	n/a	
Bankhead	Antoine	2006	Y	n/a	n/a	Identification	Co-defendant	Unlikely	n/a	
Barnes	Steven	2009	Y	n/a	n/a	Exclusion	Victim	n/a	n/a	
Batchelor	Corey	2018	Y	n/a	n/a	Exclusion	Exoneree	n/a	High	
Beaman[1]	Alan W.	2009	Y	n/a	n/a	Identification	Exoneree	Unlikely	n/a	
Beaver	Antonio	2007	Y	n/a	n/a	Exclusion	Exoneree	n/a	High	
Belcher	Michael	2001	N	I-F	Exoneree	n/a	n/a	n/a	n/a	
Berry	DeMarlo	2017	Y	n/a	n/a	Exclusion	Exoneree	n/a	Medium	
Bibbins	Gene	2003	N	I-E	Exoneree	n/a	n/a	n/a	n/a	
bin Wahad	Dhoruba	1995	Y	n/a	n/a	Identification	Exoneree	Unlikely	n/a	
Blackmon	James	2019	N	II-D	Alternate Suspect(s)	n/a	n/a	n/a	n/a	
Bonner	Samuel	2019	Y	n/a	n/a	Identification	Exoneree	Unlikely	n/a	

Last Name	First Name	Exoneration Year	Const. Report?	Error Type (if inconst.)	POI *Final* Report Pertained to (if inconst.)	Report Type (if const.)	POI Report Pertained to (if const.)	Likelihood of erroneous identification (if appl.)	Exculpatory strength (if appl.)	Effect?
Boots	Christopher	1995	N	II-D	Alternate Suspect(s)	n/a	n/a	n/a	n/a	
Bowman	James	2001	Y	n/a	n/a	Identification	Co-defendant	Unlikely	n/a	
Bozella	Dewey	2009	Y	n/a	n/a	Identification	Alternate Suspect(s)	n/a	High	
Braseel	Adam	2021	N	II-D	Alternate Suspect(s)	n/a	n/a	n/a	n/a	
Breland	Chad	2021	N	II-F	Informant	n/a	n/a	n/a	n/a	
Bridges	Timothy	2016	Y	n/a	n/a	Exclusion	Exoneree	n/a	High	
Brodie	Stephen	2010	N	II-F	Alternate Suspect(s)	n/a	n/a	n/a	n/a	
Brooks	Elvis	2022	N	I-F	Exoneree	n/a	n/a	n/a	n/a	
Brown	Sherwood	2021	Y	n/a	n/a	Exclusion	Exoneree	n/a	Medium	
Brown	Leon	2014	N	II-G	Alternate Suspect(s)	n/a	n/a	n/a	n/a	
Browning	Paul	2020	Y	n/a	n/a	Identification	Exoneree	Unlikely	n/a	
Burkhart	Richard	2017	Y	n/a	n/a	Identification	Alternate Suspect(s)	n/a	Medium	
Bush	Gary	2018	Y	n/a	n/a	Exclusion	Exoneree	n/a	High	
Cacy	Sonia	2017	Y	n/a	n/a	Identification	Exoneree	Unlikely	n/a	
Cain	Jeremiah	2022	Y	n/a	n/a	Exclusion	Co-defendant	n/a	n/a	
Camm	David	2013	Y	n/a	n/a	Identification	Co-defendant	Unlikely	n/a	
Campbell	Lori	2007	Y	n/a	n/a	Exclusion	Exoneree	n/a	High	
Canen	Lana	2012	N	I-B	Exoneree	n/a	n/a	n/a	n/a	
Carmon	Darron	2022	N	I-G	Exoneree	n/a	n/a	n/a	n/a	
Carter	Joseph	2021	Y	n/a	n/a	Identification	Alternate Suspect(s)	n/a	Low	
Carter	Edward	2010	N	II-G	Alternate Suspect(s)	n/a	n/a	n/a	n/a	
Carver	Mark	2022	N	I-E	Exoneree	n/a	n/a	n/a	n/a	
Casciaro	Mario	2016	Y	n/a	n/a	Identification	Alternate Suspect(s)	n/a	High	
Chandler	Edwin	2009	N	II-D	Alternate Suspect(s)	n/a	n/a	n/a	n/a	

Last Name	First Name	Exoneration Year	Const. Report?	Error Type (if inconst.)	POI *Final* Report Pertained to (if inconst.)	Report Type (if const.)	POI Report Pertained to (if const.)	Likelihood of erroneous identification (if appl.)	Exculpatory strength (if appl.)	Effect?
Chaney	Steven	2019	Y	n/a	n/a	Identification	Exoneree	Unlikely	n/a	
Choy[2]	Frances	2020	Y	n/a	n/a	Identification	Alternate Suspect(s)	n/a	Low	
Clark	Jeffrey	2018	Y	n/a	n/a	Identification	Victim	n/a	n/a	
Clark, Jr.	Royal	2019	N	II-E	Alternate Suspect(s)	n/a	n/a	n/a	n/a	
Cole	Timothy B.	2009	Y	n/a	n/a	Exclusion	Exoneree	n/a	Low	
Coulston	Troy	2021	N	II-D	Unconnected Person(s)	n/a	n/a	n/a	n/a	
Courteau	Paul	1999	N	I-F	Exoneree	n/a	n/a	n/a	n/a	
Cowans	Stephan	2004	N	I-B	Exoneree	n/a	n/a	n/a	n/a	
Crawford	Steven	2002	Y	n/a	n/a	Identification	Exoneree	Unlikely	n/a	
Credell	Cory	2012	Y	n/a	n/a	Identification	Exoneree	Unlikely	n/a	
Cromedy	McKinley	1999	Y	n/a	n/a	Exclusion	Exoneree	n/a	Medium	
Croy	Norma Jean	1997	Y	n/a	n/a	Identification	Co-defendant	Unlikely	n/a	
Croy	Patrick	2004	Y	n/a	n/a	Identification	Exoneree	Unlikely	n/a	
Daidone	Albert	1999	N	I-C	Exoneree	n/a	n/a	n/a	n/a	
Dandridge	Beniah Alton	2015	N	I-B	Victim	n/a	n/a	n/a	n/a	
Daniels	Elmer	2018	N	I-F	Exoneree	n/a	n/a	n/a	n/a	
Davis	Jeramie	2013	N	II-D	Alternate Suspect(s)	n/a	n/a	n/a	n/a	
Dealba, Jr.	Ignacio	2022	Y	n/a	n/a	Exclusion	Exoneree	n/a	High	
Dean	James	2009	Y	n/a	n/a	Exclusion	Exoneree	n/a	Medium	
DeJesus	George	2022	Y	n/a	n/a	Identification	Victim	n/a	n/a	
DeJesus	Melvin	2022	Y	n/a	n/a	Identification	Victim	n/a	n/a	
Dilosa	Douglas	2003	N	IV-F	Alternate Suspect(s)	n/a	n/a	n/a	n/a	
Dixon	Donald	2001	Y	n/a	n/a	Identification	Co-defendant	Unlikely	n/a	
Dombrowski	Peter	1990	N	I-F	Exoneree	n/a	n/a	n/a	n/a	

Last Name	First Name	Exoneration Year	Const. Report?	Error Type (if inconst.)	POI *Final* Report Pertained to (if inconst.)	Report Type (if const.)	POI Report Pertained to (if const.)	Likelihood of erroneous identification (if appl.)	Exculpatory strength (if appl.)	Effect?
Dorotik	Jane	2022	Y	n/a	n/a	Identification	Exoneree	Unlikely	n/a	
DuBoise	Robert	2020	Y	n/a	n/a	Exclusion	Exoneree	n/a	Medium	
Dwight	Nathan Christopher	2013	Y	n/a	n/a	Exclusion	Exoneree	n/a	Medium	
Ellis	Sean	2018	Y	n/a	n/a	Identification	Co-defendant	Maybe	n/a	
Ezell	LaShawn	2017	N	II-D	Alternate Suspect(s)	n/a	n/a	n/a	n/a	
Faison	Anthony	2001	N	II-D	Alternate Suspect(s)	n/a	n/a	n/a	n/a	
Fappiano[3]	Scott	2006	Y	n/a	n/a	Exclusion	Exoneree	n/a	Medium	
Faulkner	David	2022	N	II-G	Alternate Suspect(s)	n/a	n/a	n/a	n/a	
Ferguson	Ryan	2013	Y	n/a	n/a	Exclusion	Exoneree	n/a	Medium	
Floyd	John	2018	N	I-F	Exoneree	n/a	n/a	n/a	n/a	
Foley	Thomas	2011	Y	n/a	n/a	Identification	Exoneree	Unlikely	n/a	
Ford	Glenn	2014	N	III-A	Co-defendant	n/a	n/a	n/a	n/a	
Fritz	Dennis	1999	N	I-B	Victim	n/a	n/a	n/a	n/a	
Fulmore	James	2016	Y	n/a	n/a	Identification	Exoneree	Unlikely	n/a	
Gantt	Timothy	2008	Y	n/a	n/a	Exclusion	Exoneree	n/a	Medium	
Gardner	Jimmie	2016	Y	n/a	n/a	Identification	Exoneree	Likely	n/a	
Garner	Terence	2002	Y	n/a	n/a	Identification	Co-defendant	Unlikely	n/a	
Gibson	Roland	1993	Y	n/a	n/a	Identification	Co-defendant	Unlikely	n/a	
Giles	James Curtis	2007	N	II-D	Alternate Suspect(s)	n/a	n/a	n/a	n/a	
Gonzalez	Kathleen	2009	Y	n/a	n/a	Exclusion	Exoneree	n/a	Medium	
Gray	David A.	1999	Y	n/a	n/a	Exclusion	Exoneree	n/a	High	
Gray	Anthony	1999	Y	n/a	n/a	Exclusion	Exoneree	n/a	Medium	
Grimes	Willie	2012	N	II-G	Alternate Suspect(s)	n/a	n/a	n/a	n/a	
Hairston	Kim	1995	Y	n/a	n/a	Identification	Exoneree	Unlikely	n/a	

Last Name	First Name	Exoneration Year	Const. Report?	Error Type (if inconst.)	POI *Final* Report Pertained to (if inconst.)	Report Type (if const.)	POI Report Pertained to (if const.)	Likelihood of erroneous identification (if appl.)	Exculpatory strength (if appl.)	Effect?
Hammons	Robert	1992	Y	n/a	n/a	Exclusion	Exoneree	n/a	High	
Hardin	Garr Keith	2018	Y	n/a	n/a	Identification	Victim	n/a	n/a	
Harper	Lafayette	2019	Y	n/a	n/a	Identification	Exoneree	Unlikely	n/a	
Heins	Chad	2007	N	I-F	Exoneree	n/a	n/a	n/a	n/a	
Hernandez	Alejandro	2006	Y	n/a	n/a	Identification	Alternate Suspect(s)	n/a	Medium	
Holloway	Daryl	2016	Y	n/a	n/a	Identification	Exoneree	Unlikely	n/a	
Holton	Rudolph	2003	Y	n/a	n/a	Identification	Exoneree	Maybe	n/a	
Hood	Tyrone	2015	Y	n/a	n/a	Identification	Exoneree	Maybe	n/a	
Horn	Vernon	2018	Y	n/a	n/a	Identification	Co-defendant	Unlikely	n/a	
Howard	Timothy B.	2003	N	I-E	Exoneree	n/a	n/a	n/a	n/a	
Howard, Jr.	Eddie Lee	2021	Y	n/a	n/a	Identification	Victim	n/a	n/a	
Humiston	Chad	2004	Y	n/a	n/a	Identification	Co-defendant	Unlikely	n/a	
Ireland	Kenneth	2009	Y	n/a	n/a	Exclusion	Exoneree	n/a	Medium	
Irons	Jonathan	2020	N	IV-F	Alternate Suspect(s)	n/a	n/a	n/a	n/a	
Islam	Khalil	2021	Y	n/a	n/a	Identification	Co-defendant	Unlikely	n/a	
Jackson	Raymond	2012	Y	n/a	n/a	Exclusion	Exoneree	n/a	Medium	
Jackson	Marquis	2018	Y	n/a	n/a	Identification	Co-defendant	Unlikely	n/a	
Jackson	Richard	2000	N	I-B	Exoneree	n/a	n/a	n/a	n/a	
James	Thomas	2022	Y	n/a	n/a	Exclusion	Exoneree	n/a	Medium	
James	Gary Lamar	2003	N	I-E	Exoneree	n/a	n/a	n/a	n/a	
Jernigan	Rachel	2008	Y	n/a	n/a	Exclusion	Exoneree	n/a	Medium	
Johnson	Timothy	2013	Y	n/a	n/a	Identification	Exoneree	Maybe	n/a	
Johnson	Bobby	2015	Y	n/a	n/a	Identification	Alternate Suspect(s)	n/a	High	
Johnson	Roderick	2020	Y	n/a	n/a	Identification	Co-defendant	Unlikely	n/a	

Last Name	First Name	Exoneration Year	Const. Report?	Error Type (if inconst.)	POI *Final* Report Pertained to (if inconst.)	Report Type (if const.)	POI Report Pertained to (if const.)	Likelihood of erroneous identification (if appl.)	Exculpatory strength (if appl.)	Effect?
Johnson	Charles	2017	N	II-D	Alternate Suspect(s)	n/a	n/a	n/a	n/a	
Jones	Wilbert	2018	Y	n/a	n/a	Identification	Alternate Suspect(s)	n/a	Medium	
Kinge	Shirley	1992	N	I-B	Exoneree	n/a	n/a	n/a	n/a	
Krone	Ray	2002	N	II-D	Alternate Suspect(s)	n/a	n/a	n/a	n/a	
Kyles	Curtis	1998	Y	n/a	n/a	Identification	Exoneree	Maybe	n/a	
LaBolt, Jr.	William	1994	N	I-B	Exoneree	n/a	n/a	n/a	n/a	
Lobato	Kirstin	2017	Y	n/a	n/a	Exclusion	Exoneree	n/a	Medium	
Long	Ronnie	2020	N	I-F	Exoneree	n/a	n/a	n/a	n/a	
Lumpkins	Todd	1989	Y	n/a	n/a	Identification	Exoneree	Unlikely	n/a	
Majors	Robert	2020	N	II-F	Co-defendant	n/a	n/a	n/a	n/a	
Martorano	Raymond	1999	N	I-C	Co-defendant	n/a	n/a	n/a	n/a	
Maxwell	Bobby Joe	2018	Y	n/a	n/a	Identification	Exoneree	Unlikely	n/a	
McCall	Corey	2021	Y	n/a	n/a	Exclusion	Exoneree	n/a	Medium	
McCollum	Claude	2007	N	II-D	Alternate Suspect(s)	n/a	n/a	n/a	n/a	
McCollum	Henry	2014	N	II-G	Alternate Suspect(s)	n/a	n/a	n/a	n/a	
McCoy	Isaiah	2017	Y	n/a	n/a	Exclusion	Exoneree	n/a	Medium	
McCoy	Troshawn	2017	N	II-D	Alternate Suspect(s)	n/a	n/a	n/a	n/a	
McIntosh	Norman	2016	N	II-D	Alternate Suspect(s)	n/a	n/a	n/a	n/a	
Merrill	Thomas	1995	Y	n/a	n/a	Identification	Alternate Suspect(s)	n/a	Low	
Mikes	Melvin	1992	Y	n/a	n/a	Identification	Exoneree	Maybe	n/a	
Miles	Richard	2012	N	II-D	Alternate Suspect(s)	n/a	n/a	n/a	n/a	
Miranda	Roberto	1996	Y	n/a	n/a	Identification	Exoneree	Maybe	n/a	
Monson	Lamarr	2017	N	II-C	Alternate Suspect(s)	n/a	n/a	n/a	n/a	
Moore	Joshua	2001	Y	n/a	n/a	Identification	Exoneree	Unlikely	n/a	

Last Name	First Name	Exoneration Year	Const. Report?	Error Type (if inconst.)	POI *Final* Report Pertained to (if inconst.)	Report Type (if const.)	POI Report Pertained to (if const.)	Likelihood of erroneous identification (if appl.)	Exculpatory strength (if appl.)	Effect?
Moran	Christopher	2022	Y	n/a	n/a	Identification	Co-defendant	Maybe	n/a	
Mozee	Stanley	2019	Y	n/a	n/a	Exclusion	Exoneree	n/a	High	
Nash	Donald	2020	N	II-G	Alternate Suspect(s)	n/a	n/a	n/a	n/a	
Nelson	Bruce	1991	Y	n/a	n/a	Identification	Co-defendant	Unlikely	n/a	
Newsome	James	1995	N	II-A	Alternate Suspect(s)	n/a	n/a	n/a	n/a	
Ngov	Matthew	2019	Y	n/a	n/a	Exclusion	Exoneree	n/a	High	
Nicholson	Mallory	2022	Y	n/a	n/a	Exclusion	Exoneree	n/a	Medium	
Nolley	John	2018	N	I-F	Exoneree	n/a	n/a	n/a	n/a	
O'Laughlin	Michael	2010	Y	n/a	n/a	Exclusion	Exoneree	n/a	Medium	
Ochoa	James	2006	Y	n/a	n/a	Exclusion	Exoneree	n/a	High	
Onyiah	Obina	2021	Y	n/a	n/a	Exclusion	Exoneree	n/a	Medium	
Palmer	Charles	2016	Y	n/a	n/a	Identification	Co-defendant	Maybe	n/a	
Penalver	Seth	2012	Y	n/a	n/a	Exclusion	Exoneree	n/a	Medium	
Perez	Josefina	2005	Y	n/a	n/a	Identification	Alternate Suspect(s)	n/a	Medium	
Philen	Randall	2012	N	II-D	Alternate Suspect(s)	n/a	n/a	n/a	n/a	
Pittman	Marcellous	2022	Y	n/a	n/a	Exclusion	Exoneree	n/a	High	
Porter	Allen Wayne	2010	N	II-D	Alternate Suspect(s)	n/a	n/a	n/a	n/a	
Prentice	Mark	1995	N	I-B	Exoneree	n/a	n/a	n/a	n/a	
Proctor	Eric	1995	N	II-D	Alternate Suspect(s)	n/a	n/a	n/a	n/a	
Qualls	Ronnie	2020	Y	n/a	n/a	Identification	Exoneree	Unlikely	n/a	
Reed	Deshawn	2017	N	II-H	Alternate Suspect(s)	n/a	n/a	n/a	n/a	
Reeder	Kuantau	2021	N	I-G	Exoneree	n/a	n/a	n/a	n/a	
Register	Kash	2013	Y	n/a	n/a	Exclusion	Exoneree	n/a	Medium	
Rios	Jaime	2022	Y	n/a	n/a	Exclusion	Exoneree	n/a	Medium	

Last Name	First Name	Exoneration Year	Const. Report?	Error Type (if inconst.)	POI *Final* Report Pertained to (if inconst.)	Report Type (if const.)	POI Report Pertained to (if const.)	Likelihood of erroneous identification (if appl.)	Exculpatory strength (if appl.)	Effect?
Rockette	Xavier	2013	Y	n/a	n/a	Identification	Exoneree	Unlikely	n/a	
Rollins, IV	Offord	1996	Y	n/a	n/a	Identification	Victim	n/a	n/a	
Royer	Andrew	2021	N	I-B	Co-defendant	n/a	n/a	n/a	n/a	
Ruiz-Cortez	Refugio	2010	Y	n/a	n/a	Exclusion	Exoneree	n/a	High	
Seri	Michael Caesar	2003	N	II-C	Alternate Suspect(s)	n/a	n/a	n/a	n/a	
Serrano	Pedro	2017	N	I-E	Exoneree	n/a	n/a	n/a	n/a	
Sharpe	Dontae	2019	Y	n/a	n/a	Exclusion	Exoneree	n/a	Medium	
Shelden	Debra	2009	Y	n/a	n/a	Exclusion	Exoneree	n/a	Medium	
Shepherd	Charles	2001	N	II-D	Alternate Suspect(s)	n/a	n/a	n/a	n/a	
Showers	Kurtis DeAngelo	1998	N	I-E	Exoneree	n/a	n/a	n/a	n/a	
Siehl	Kevin	2016	N	III-B	Exoneree	n/a	n/a	n/a	n/a	
Siller	Thomas	2011	Y	n/a	n/a	Identification	Exoneree	Unlikely	n/a	
Simmons III	Tommy	2003	Y	n/a	n/a	Identification	Victim	n/a	n/a	
Sledge	Joseph	2015	N	I-F	Exoneree	n/a	n/a	n/a	n/a	
Smith	Michael	2009	Y	n/a	n/a	Exclusion	Exoneree	n/a	Medium	
Smith	Kenneth	2021	Y	n/a	n/a	Exclusion	Exoneree	n/a	Medium	
Smith	Jon Keith	2000	Y	n/a	n/a	Identification	Exoneree	Unlikely	n/a	
Sonnier	Ernest	2018	Y	n/a	n/a	Identification	Alternate Suspect(s)	n/a	Medium	
Strickland	Kevin	2021	N	I-E	Exoneree	n/a	n/a	n/a	n/a	
Styles	Larod	2017	N	II-D	Alternate Suspect(s)	n/a	n/a	n/a	n/a	
Sullivan	Michael	2019	Y	n/a	n/a	Identification	Co-defendant	Unlikely	n/a	
Taylor	Ada JoAnn	2009	Y	n/a	n/a	Exclusion	Exoneree	n/a	Medium	
Thomas	Marvin	1993	Y	n/a	n/a	Identification	Victim	n/a	n/a	
Thompson	Luqris	2012	Y	n/a	n/a	Identification	Co-defendant	Unlikely	n/a	

Last Name	First Name	Exoneration Year	Const. Report?	Error Type (if inconst.)	POI *Final* Report Pertained to (if inconst.)	Report Type (if const.)	POI Report Pertained to (if const.)	Likelihood of erroneous identification (if appl.)	Exculpatory strength (if appl.)	Effect?
Tillman	Michael	2010	Y	n/a	n/a	Identification	Co-defendant	Maybe	n/a	
Tomlin	Charles	1994	Y	n/a	n/a	Exclusion	Exoneree	n/a	High	
Turner	Kenneth	1995	Y	n/a	n/a	Identification	Exoneree	Unlikely	n/a	
Vanderhorst	Zachary	2021	Y	n/a	n/a	Identification	Exoneree	Unlikely	n/a	
Velez	John	2017	Y	n/a	n/a	Exclusion	Exoneree	n/a	Medium	
Virgil	William	2017	Y	n/a	n/a	Identification	Exoneree	Unlikely	n/a	
Walker	Quedillis Ricardo	2003	Y	n/a	n/a	Identification	Co-defendant	Unlikely	n/a	
Warney	Douglas	2006	N	II-D	Alternate Suspect(s)	n/a	n/a	n/a	n/a	
Warney[4]	Douglas	2006	N	I-C	Exoneree	n/a	n/a	n/a	n/a	
Washington	Wayne	2015	Y	n/a	n/a	Identification	Co-defendant	Maybe	n/a	
Waters	Kenneth	2001	N	I-F	Exoneree	n/a	n/a	n/a	n/a	
Whirl	Shawn	2015	Y	n/a	n/a	Identification	Exoneree	Unlikely	n/a	
White	Joseph	2008	Y	n/a	n/a	Exclusion	Exoneree	n/a	Medium	
Wiggins	David Lee	2012	Y	n/a	n/a	Exclusion	Exoneree	n/a	Medium	
Williams	James Curtis	2012	Y	n/a	n/a	Exclusion	Exoneree	n/a	Medium	
Williams	Barry	2021	Y	n/a	n/a	Exclusion	Exoneree	n/a	Medium	
Williams	Archie	2019	N	II-G	Alternate Suspect(s)	n/a	n/a	n/a	n/a	
Williams	Christopher	2019	N	II-D	Unconnected Person(s)	n/a	n/a	n/a	n/a	
Williams	Christopher	2021	N	II-D	Unconnected Person(s)	n/a	n/a	n/a	n/a	
Williams	Michael Anthony	2005	Y	n/a	n/a	No Value	n/a	n/a	n/a	
Williams	Terrance	2020	Y	n/a	n/a	Identification	Exoneree	Unlikely	n/a	
Williamson	Ronald Keith	1999	N	I-B	Victim	n/a	n/a	n/a	n/a	
Winslow	Thomas	2009	Y	n/a	n/a	Exclusion	Exoneree	n/a	Medium	
Wirkkala	Luke	2021	Y	n/a	n/a	Identification	Exoneree	Unlikely	n/a	

Last Name	First Name	Exoneration Year	Const. Report?	Error Type (if inconst.)	POI *Final* Report Pertained to (if inconst.)	Report Type (if const.)	POI Report Pertained to (if const.)	Likelihood of erroneous identification (if appl.)	Exculpatory strength (if appl.)	Effect?
Woodworth	Mark	2014	Y	n/a	n/a	Identification	Exoneree	Unlikely	n/a	
Zapata	Rodrigo	2001	Y	n/a	n/a	Identification	Exoneree	Unlikely	n/a	
Zimmer	Walter	2011	Y	n/a	n/a	Identification	Exoneree	Unlikely	n/a	

[1]Fingerprints recovered from clock radio identified victim's current boyfriend, the exoneree, and an unidentifiable source. Identification to exoneree is more implicating than an inconclusive report.
[2]Exoneree's prints were not on any items submitted for fingerprint testing. Prints did match an alternate suspect. The identification to an alternate suspect is more exculpatory for the exoneree than an exclusion report that excludes the exoneree.
[3]Prints found at crime scene identified the victim but excluded the exoneree. This exclusion report is more probative of innocence than the identification report.
[4]This case violates our coding procedure of one-case-one-code, however, we chose to count this case twice in accordance with Cole & Scheck's coding procedure for consistency.

Key

- Incriminated defendant
- Exculpated defendant
- Neutral or ambiguous effect on defendant
